# A Guide to the Practical Examination of Urine

**Published:** 1874-12

**Authors:** 


*A Guide to the Practical Examination of the Urine. For the use Physicians and Students.* By James Tyson, M. D. Philadelphia: Lindsay & Blakiston, 1875.

This little work is devoted to a subject upon which physicians and students are as a rule, but poorly informed. This being the case, the question arises, is this work capable of imparting the desired information? To this there seems to be to us, no danger in replying in the affirmative.

Of a convenient size, and embracing all of the practical points in urinary analysis it will, we have no doubt be found to meet the wants of those, both Students and Practitioners, who feel a desire to investigate the more common points in this subject. The work is conveniently subdivided into sections and fully indexed. The illustrations, thirty in number, are, some of them borrowed from other writers, but in a larger number of cases are original. Dr. Tyson has introduced in this work the German method of approximate estimate which we do not remember to have seen in any other English work.

